# Laccase‐Based Self‐Amplifying Catalytic System Enables Efficient Antibiotic Degradation for Sustainable Environmental Remediation

**DOI:** 10.1002/advs.202300210

**Published:** 2023-05-21

**Authors:** Ying Xia, Liming Xia, Xinda Lin

**Affiliations:** ^1^ Key Laboratory of Bioorganic Synthesis of Zhejiang Province College of Biotechnology and Bioengineering Zhejiang University of Technology Hangzhou 310014 P. R. China; ^2^ Key Laboratory of Biomass Chemical Engineering of Ministry of Education College of Chemical and Biological Engineering Zhejiang University Hangzhou 310027 P. R. China

**Keywords:** antibiotic, bioremediation, laccase, lignocellulose

## Abstract

Antibiotic contamination poses potential risks to ecosystems and human health. Laccase (LAC) has emerged as a promising biocatalyst for the oxidation of environmentally toxic contaminants with high catalytic efficiency; however, its large‐scale application is hindered by enzyme costs and dependency on redox mediators. Herein, a novel self‐amplifying catalytic system (SACS) for antibiotic remediation that does not require external mediators is developed. In SACS, a natural mediator‐regenerating koji with high‐activity LAC, derived from lignocellulosic waste, initiates the chlortetracycline (CTC) degradation. Subsequently, an intermediate product, CTC327, identified as an active mediator for LAC via molecular docking, is formed and then starts a renewable reaction cycle, including CTC327‐LAC interaction, stimulated CTC bioconversion, and self‐amplifying CTC327 release, thus enabling highly efficient antibiotic bioremediation. In addition, SACS exhibits excellent performance in producing lignocellulose‐degrading enzymes, highlighting its potential for lignocellulosic biomass deconstruction. To demonstrate its effectiveness and accessibility in the natural environment, SACS is used to catalyze in situ soil bioremediation and straw degradation. The resulting CTC degradation rate is 93.43%, with a straw mass loss of up to 58.35% in a coupled process. This mediator regeneration and waste‐to‐resource conversion in SACS provides a promising route for environmental remediation and sustainable agricultural practices.

## Graphical abstract

Efficient removal of antibiotics in soils is enabled by the self‐amplifying catalytic system (SACS). SACS is established via three steps: natural mediator regeneration during straw degradation by a laccase (LAC)‐producing consortium, an active intermediate release, and LAC–intermediate interaction. This low‐cost and environmentally friendly catalytic system allows for organic pollutant remediation and lignocellulosic biomass degradation without the need for additional mediators.

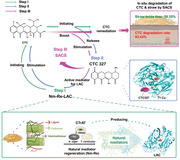

## 1 Introduction

Antibiotic accumulation in agricultural soils poses a potential risk to the soil ecosystem and crop growth, and ultimately threatens human health.^[^ ^1^ ^]^ Tetracycline antibiotics (TCs), such as chlortetracycline (CTC), oxytetracycline (OTC), and tetracycline (TTC), are broad‐spectrum antimicrobial agents widely present in soils. In general, TCs are hard to be completely metabolized in vivo, and thus large proportions are excreted into the ecosystem via feces or urine.^[^ ^2^ ^]^ TC residues not only increase bacterial resistance but also aggravate the proliferation of antibiotic‐resistance genes.^[^ ^3^ ^]^ Considering the potential ecological and environmental risks, actions should be taken to detoxify antibiotics in various environmental systems including soil.

Many research efforts have focused on the removal of TCs from wastewater,^[^ ^4^ ^]^ but the remediation of TC‐contaminated soil, particularly agricultural soil, has received less attention.^[^ ^5^ ^]^ Overall, the natural biodegradation of TCs mediated by native microorganisms in the soil is comparatively slow.^[^ ^6^ ^]^ In recent years, efforts focusing on bioremediation have been made to accelerate the TC degradation process in soil,^[^ ^6^ ^]^ such as biochar,^[^ ^7^ ^]^ indigenous bacterial strains,^[^ ^8^ ^]^ and a microalgae–bacteria consortium.^[^ ^7b^ ^]^

Nevertheless, the large‐scale application of these strategies is limited by several shortcomings such as low efficiency, high cost, and long treatment cycles.^[^ ^9^ ^]^ Moreover, their application to soil remediation is hindered by the complexity of the soil environment.^[^ ^10^ ^]^ Therefore, it is imperative to develop effective and economical bioremediation strategies to enhance the removal of TCs in agricultural soils.

Bioremediation based on laccases (EC 1.10.3.2, LAC) is an environmentally friendly approach for detoxifying persistent organic pollutants.^[^ ^11^ ^]^ LAC belongs to the multicopper oxidase family and catalyzes phenolic compound oxidation. The catalytic core of LAC is organized into two copper centers. The T1 copper site (T1 Cu) is the primary electron acceptor. After accepting a single electron, T1 Cu transfers the electron to the T2/T3 trinuclear copper center (T2/T3 Cu), accompanied by the reduction of O_2_ to H_2_O. The difference in the redox potential between T1 Cu and the substrate is known as the electrochemical driving force of electron transfer and plays a crucial role in the oxidation efficiency of LAC.^[^ ^12^ ^]^ Owing to their broad substrate specificity, LACs have been applied for the bioremediation of TCs,^[^ ^13^ ^]^ dyes,^[^ ^14^ ^]^ pesticides,^[^ ^15^ ^]^ polycyclic aromatic hydrocarbons,^[^ ^16^ ^]^ and endocrine disrupters.^[^ ^17^ ^]^ However, the application of LAC is hindered because its redox potential is comparatively low (0.4–0.8 V) and thus LAC is incapable of oxidizing substrates with high redox potentials.^[^ ^12^ ^]^ Several low‐molecular–weight phenolic compounds, termed mediators, have been reported to assist LAC in oxidation by producing active free radicals and are considered key players in enhancing the oxidation efficiency of LAC.^[^ ^18^ ^]^ Although the LAC‐mediator system has great potential in the area of bioremediation, its feasibility is restricted due to the high cost of LAC as well as mediators.

**Figure 1:**
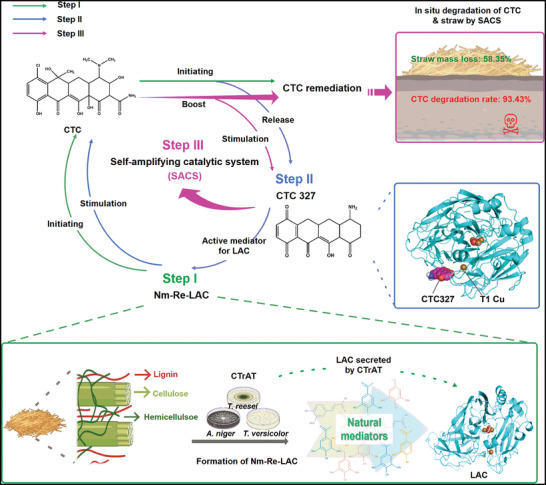
Schematic overview of the three‐step development of the self‐amplifying catalytic system (SACS). Natural mediator‐regenerating koji with high laccase activity (Nm‐Re‐LAC) first initiated chlortetracycline (CTC) degradation (Step I) and then released the active intermediate CTC327 working as a mediator for laccase (LAC) (Step II). This LAC‐CTC327 interaction stimulated a self‐amplifying chain reaction, which led to enhanced CTC remediation efficiency (Step III).

Here we developed a novel self‐amplifying catalytic system (SACS) using inexpensive lignocellulosic sources to address the aforementioned challenges. The design of this SACS eliminated the need for redox mediators. Instead, SACS is based on a natural mediator‐regenerating koji with high LAC activity (Nm‐Re‐LAC), which was prepared by solid‐state fermentation of rice straw via a fungal consortium (named CTrAT) comprising *Trichoderma reesei* (*T. reesei*) ZJ09, *Aspergillus niger* (*A. niger*), and *Trametes versicolor* (*T. versicolor*). SACS could be established via three steps (**Figure** **1**). First, Nm‐Re‐LAC initiated the biodegradation of CTC. Second, a CTC‐derived degradation intermediate (CTC327), also identified as an active mediator for LAC, was formed. Third, the interactions between CTC327 and LAC started a chain reaction toward the highly efficient conversion of CTC. In addition to antibiotic remediation, SACS was capable of simultaneous secretion of multiple lignocellulose‐degrading enzymes, thus enabling the synergistic degradation of lignocellulosic biomass. Therefore, we also assayed the performance of SACS in the biodegradation of both TCs in soil and agricultural waste via a coupling process. This work provides an efficient and low‐cost catalytic system for the organic pollutant remediation and the lignocellulosic biomass degradation.

## 2 Results

### 2.1 Tetracycline Antibiotic Degradation Mediated by the LAC‐Mediator System

The antibiotic degradation performance of different LAC‐mediator systems was investigated. The target TCs (OTC and CTC) selected here are the two major residual antibiotics in the soil that are relatively recalcitrant to degradation. The LAC used here was produced by the *T. reesei* ZJ09.^[^ ^19^ ^]^ As shown in Table S1, Supporting Information, the widely applied synthetic mediators 2,2′‐azino‐bis(3‐ethylbenzothiazoline‐6‐sulfonic acid (ABTS) and 1‐hydroxybenzotriazole (HBT) could assist LAC in the degradation of tetracycline antibiotics. After 4h‐treatment, the LAC‐ABTS system achieved degradation rates of 46.73% and 86.10% for OTC and CTC, respectively, and those of the HBT‐mediated treatments were 30.31% and 90.25%, respectively.

Despite their confirmed efficiency, artificial mediators can result in additional costs and LAC inactivation, owing to their potential toxicity.^[^ ^20^ ^]^ In contrast, natural mediators, such as syringaldehyde (Syr) and vanillin (Van), are more environmentally friendly.^[^ ^21^ ^]^ The removal rates of CTC using LAC‐Syr and LAC‐Van systems were 71.43% and 75.80%, respectively (Table S1, Supporting Information). Notably, an enhanced degradation rate is observed when Syr and Van are combined. The LAC‐Syr/Van system degraded 90.52% of the OTC and 82.03% of the CTC. This result indicates that the coexistence of multiple natural mediators could lead to a cooperative effect in LAC‐mediator systems, which could be the result of the chain reactions among multiple radical species.^[^ ^21^ ^]^

During the lignin biodegradation process, various phenolic compounds related to lignin polymer could be released, forming a mixture of natural mediators for LAC.^[^ ^21^ ^]^ Electron paramagnetic resonance (EPR) spectroscopy was used to monitor the natural mediator formation during LAC‐catalyzed lignin degradation. The oxidation of mediators is presumed to produce radical intermediates.^[^ ^22^ ^]^ Thus, the higher radical concentration achieved in the reaction system could be ascribed to a higher concentration of small, soluble radical compounds with function as mediators. As shown in Figure S1, Supporting Information, the radicals were detected during this process, which indicated the LAC‐catalyzed oxidation was fast enough to generate a net positive formation of radicals. The highest radical concentration reached 67 µm after 1 h of LAC treatment. Therefore, taking full advantage of this lignin‐derived phenolic compounds mixture could be a promising strategy to boost the catalytic capacity of LAC toward recalcitrant aromatic compounds of environmental interest, including antibiotics.

### 2.2 Nm‐Re‐LAC‐Mediated Antibiotic Degradation

Lignin is resistant to degradation owing to its complex and rigid structure, which affects the effective release of redox mediators. Although the *T. reesei* ZJ09 can produce a series of lignocellulose‐degrading enzymes (i.e., cellulase, xylanase, and LAC) and possesses notable catalytic activity toward lignocellulosic biomass, the current strain has two drawbacks. First, despite the partial degradation of lignin in straw, the efficiency is still insufficient to achieve efficient lignin oxidization. Second, the *β*‐glucosidase activity (BGA) in the cellulase complex produced by *T. reesei* ZJ09 is extremely low,^[^ ^23^ ^]^ which results in the end‐product inhibition of cellulases.^[^ ^24^ ^]^

Here, to construct an a mediator LAC‐natural mediator system for tetracycline antibiotics degradation, we first developed a fungal consortium that could effectively degrade straw (named CTrAT). Subsequently, koji formed via CTrAT‐mediated straw degradation, which contained high‐activity LAC and various phenolic derivatives, was used for antibiotic removal.

**Figure 2:**
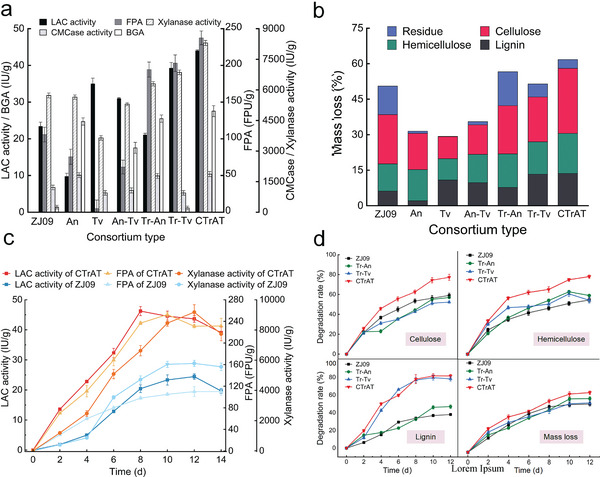
Enzyme production and straw lignocellulose degradation by the fungal consortium. a) Enzyme activities of the individual fungus and fungal consortium using rice straw as substrate. All samples were collected on day 10. b) Mass loss and composition of straw after a 10 days treatment by the individual fungus and fungal consortium. c) Changes in lignocellulolytic enzyme activities of CTrAT and *Trichoderma reesei* ZJ09 during a solid‐state fermentation (SSF) process. d) Degradation rates of cellulose, hemicellulose, lignin, and mass loss during fungal consortium treatment. ZJ09 refers to the recombinant *T. reesei* with *Pycnoporus sanguineus* LAC gene, An refers to *Aspergillus niger*, Tv refers to *Trametes versicolor*, Tr–An refers to the consortium of ZJ09 and *A. niger*, Tr–Tv refers to the consortium of ZJ09 and *T. versicolor*, An–Tv refers to the consortium of *A. niger* and *T. versicolor*, CTrAT refers to the consortium of ZJ09, *A. niger*, and *T. versicolor*. Error bars represent S.D. (*n* = 3).

First, to fully alleviate the recalcitrance of straw, especially the lignin, and release of lignin‐derived natural mediators, a straw‐degrading fungal consortium was constructed by using *T. reesei* ZJ09, *Aspergillus niger* (*A. niger*), and *Trametes versicolor* (*T. versicolor*). *A. niger* is a well‐exploited producer for high‐activity *β*‐glucosidase with desirable enzymatic properties.^[^ ^25^ ^]^ As shown in **Figure** **2**a, the introduction of *A. niger* boosted BGA as well as the endoglucanase activity (CMCase activity) by the consortium of *T. reesei* ZJ09 and *A. niger* (Tr–An), and the BGA and CMCase activity reached 25.49 and 1778.72 IU g^−1^, respectively, which was 18.6‐ and 1.47–fold higher, respectively, than that of *T. reesei* ZJ09 alone. Accompanied by the BGA enhancement, feedback inhibition caused by cellobiose accumulation could be relieved, leading to an improvement in total cellulase activity (FPA). FPA produced by the Tr–An increased to 194.32 FPU g^−1^, which was 1.84‐fold higher than that of *T. reesei* ZJ09 alone. However, the rate of lignin degradation by this fungi combination remains low because of deficient LAC production.

*T. versicolor* has a good capability for LAC production and thus can enable the efficient processing of lignin which *T. reesei* and *A. niger* fall short of.^[^ ^26^ ^]^ Furthermore, besides LAC, *T. versicolor* can secrete several other lignin‐degrading enzymes targeting a variety of phenolic organic pollutants,^[^ ^27^ ^]^ and is therefore of particular interest for environmental applications. Although BGA and CMCase activities exhibited mild decreases, the combination of *T. reesei* ZJ09 and *T. versicolor* (Tr–Tv) significantly enhanced LAC production. The LAC activity was 1.68‐fold higher to 39.21 IU g^−1^ compared to that of *T. reesei* ZJ09 alone (Figure 2a), and the degradation rate of lignin increased significantly by 2.16‐fold (Figure 2b). However, the final mass loss did not improve, which could be ascribed to decreased CMCase activity (Figure 2a,b). As shown in Figure 2, although *A. niger* and *T. versicolor* were superior to *T. reesei* in producing *β*‐glucosidase and LAC, respectively, the consortium of *A. niger* and *T. versicolor* (An–Tv) does not result in higher straw degradation ability. On day 10 of SSF, the overall biomass loss achieved with An–Tv was lower than that achieved with *T. reesei* ZJ09.

The results of enzyme production and straw degradation by a consortium of three fungi (*T. reesei* ZJ09, *A. niger*, and *T. versicolor*), designated as CTrAT, indicated that this consortium had good compatibility and could overcome the deficiencies of the two co‐cultured strains. The FPA and LAC activities produced by CTrAT exhibited rapid growth within the initial 2 days. The LAC activity, FPA, xylanase activity, CMCase activity, and BGA were 1.88‐, 2.25‐, 1.45‐, 1.54‐, and 20.10‐fold, respectively, on day 10 than those of *T. reesei* ZJ09 alone (Figure 2c). Accordingly, CTrAT improved straw decomposition with higher lignin, cellulose, hemicellulose, and biomass degradation rates (Figure 2). The rapid degradation of cellulose and hemicellulose by CTrAT occurred at the start of the SSF stage (0–4 days), and then the degradation rate slowed (Figure 2d). On day 8, the degradation of cellulose and hemicellulose had been accelerated again with the LAC activity topping 46.23 IU g^−1^ (Figure 2c), reaching 77.25% and 75.33%, respectively, on day 12. One explanation is that efficient lignin decomposition mediated by the improved secretion of LAC by CTrAT, subsequently made cellulose and hemicellulose more readily available to microorganisms. On day 10 of SSF, the lignin degradation rate of CTrAT was to 81.64%, which was significantly higher than that of *T. reesei* ZJ09 (36.81%). The overall biomass loss achieved by CTrAT was 63.29% on day 12, 1.24‐fold higher than that of *T. reesei* ZJ09.

During the effective straw degradation process mediated by CTrAT, natural mediator‐regenerating koji with high LAC activity (Nm‐Re‐LAC) was formed, in which the phenolic compounds released by lignin decomposition could serve as natural mediators for LAC. After 4 h‐treatment, without the addition of any mediator, the Nm‐Re‐LAC‐mediated degradation rates of TCs reached 89.12% for OTC and 95.69% for CTC, comparable to those of suitable LAC‐mediator systems (Table S1, Supporting Information). Similarly, Nm‐Re‐LAC completely eliminated the growth inhibition of different types of microbial communities caused by TCs (Table S2, Supporting Information). CTC was chosen for subsequent studies because it had a comparatively higher degradation rate and sensitivity after Nm‐Re‐LAC treatment in our screening.

**Figure 3:**
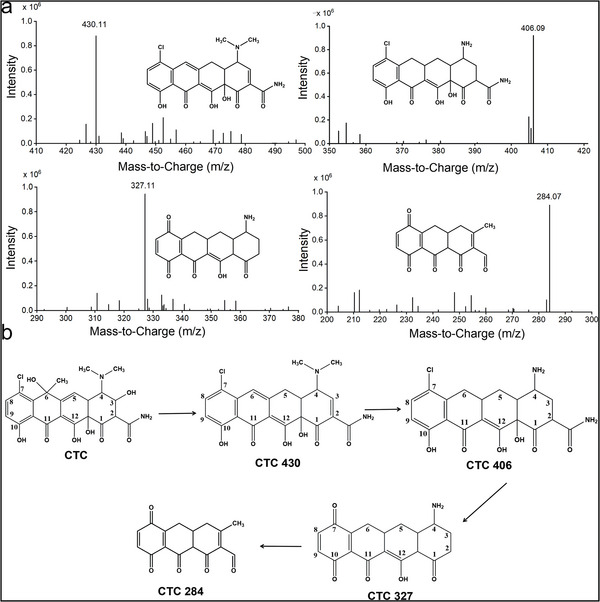
Reaction pathway analyses of chlortetracycline (CTC) catalyzed by natural mediator‐regenerating koji with high laccase activity (Nm‐Re‐LAC). a) Mass spectra of CTC transformation products after Nm‐Re‐LAC treatment. CTC (80 mg L^−1^) was treated using Nm‐Re‐LAC at a pH of 4.0 and temperature at 50 °C with 0.2 IU L^−1^ LAC. b) Proposed degradation pathway of CTC.

Phenolic xenobiotics can be transformed into less toxic compounds after treatment with LAC‐mediator systems.^[^ ^28^ ^]^ In this study, four possible biodegradation intermediates of CTC after Nm‐Re‐LAC treatment, with measured mass (m/z) 430.11, 406.09, 327.11, and 284.07 (**Figure** **3**a), were identified, while the m/z of CTC was 480.13. These four transformation products were designated CTC 430, CTC 406, CTC 327, and CTC 284. A mechanism of CTC transformation by Nm‐Re‐LAC was proposed based on the four identified products (Figure 3b).

CTC was first demethylated and dehydroxylated to form CTC 430, and then the dimethyl amino group at the C4 position was bi‐demethylated to form CTC 406. Subsequently, a phenoxy radical was generated after the dechlorination (—Cl) reaction at C7 and oxidized to the corresponding quinone (CTC 284). A similar metabolic pathway was suggested by Pulicharla et al.^[^ ^29^ ^]^ and reported that the LAC‐catalyzed degradation of CTC mainly included hydrogenation, dehydroxylation, demethylation, decarboxylation, and deamination reactions. Here, we identified new intermediates of CTC degradation, filling a gap in the mechanism of CTC degradation by fungal active koji. As shown in Figure 3, in the benzene ring of CTC, a chlorine atom replaces a hydrogen atom in the hydroxyl group. It has been reported that additional chlorine atoms enhance fat solubility, leading to increased CTC toxicity.^[^ ^8^ ^]^ Our results revealed that CTC was dechlorinated during the degradation process, indicating that the bio‐catalytic system mediated by Nm‐Re‐LAC could achieve a good detoxification effect.

### 2.3 Self‐Amplifying Catalytic System Based on Nm‐Re‐LAC

In this study, two quinoid compounds (CTC327 and CTC284) were generated via Nm‐Re‐LAC‐catalyzed CTC degradation (Figure 3). Quinones are suitable electron donors in the redox processes,^[^ ^30^ ^]^ thereby promoting the remediation efficiency of contaminants.^[^ ^31^ ^]^ Quinone‐based compounds have been reported to improve the decolorization rates of multiple azo dyes.^[^ ^32^ ^]^

To elucidate the catalysis‐enhancing mechanism between LAC and quinoid compounds, the interactions between *Pycnoporus sanguineus* (*P. sanguineus*) LAC and the major CTC degradation products (CTC430, CTC406, CTC327, and CTC284), as well as the typical artificial mediator (HBT) and natural mediators (syringaldehyde and vanillin), were investigated using molecular docking. As shown in Table S3, Supporting Information, the affinity value of CTC327 and LAC at the T1 Cu, one of the catalytic cores of LAC, was −6.4 kcal mol^−1^, while that of HBT and LAC was −4.8 kcal mol^−1^, showing the interaction between CTC327 and LAC could occur spontaneously and CTC327 had relatively higher affinity compared to HBT. In addition, CTC327 reduced the T1 Cu‐ligation distance compared to the other mediators (Table S3, Supporting Information). These results indicated that an intermediate, CTC327, was produced during CTC degradation catalyzed by Nm‐Re‐LAC, which could act as an active mediator for LAC.

**Figure 4:**
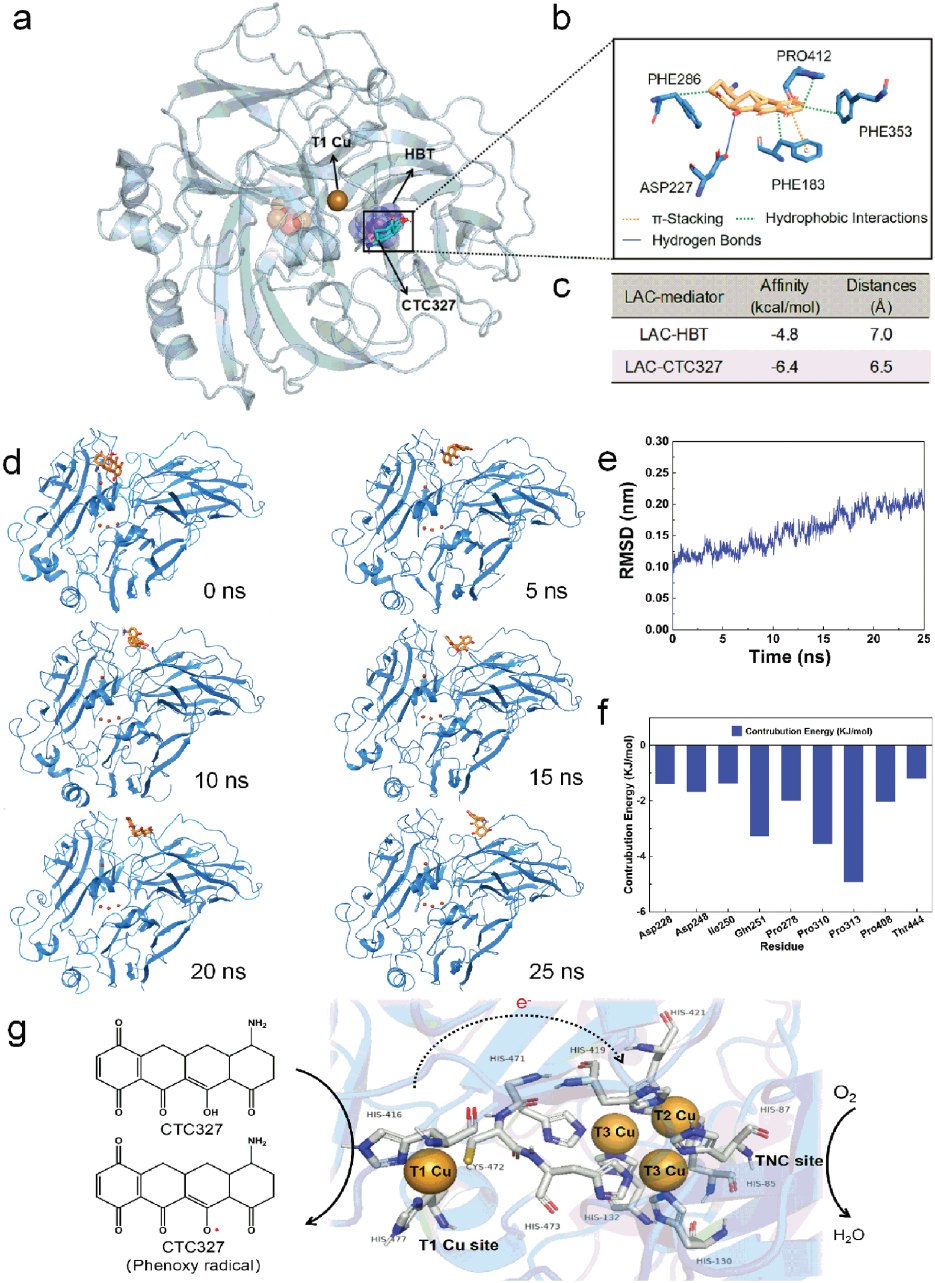
Molecular docking and dynamics simulations of LAC–CTC327. a) The binding pocket of LAC‐CTC327 and LAC‐HBT at T1 Cu site. b) Interactions between laccase (LAC) and CTC327 within 5 Å distance. c) LAC‐mediator complex geometry at T1 Cu site. Distance refers to the average distance between the nuclei of T1 Cu and atoms in mediators. d) Snapshots of molecular dynamics simulation of LAC with CTC327. e) RMSD graph of LAC docked with CTC327. f) The residue‐wise binding energy decomposition of LAC‐CTC327. g) Predicted electron transfer mechanism of LAC‐CTC327.

Thus, we established a three‐step SACS (Figure 1). In SACS, after the initial CTC degradation mediated by Nm‐Re‐LAC, the active mediator CTC327 was produced. Subsequently, the interactions between CTC327 and LAC could stimulate the remediation of CTC and in turn increase the release of CTC327, thus creating a chain reaction for high‐efficiency CTC remediation. The interactions between LAC and CTC327 were analyzed to elucidate the mechanism of action of SACS. In the LAC‐CTC327 complex, CTC327 was surrounded by the residues Pro412, Phe286, Phe353, Phe183, and Asp227 (**Figure** **4**b). In the binding pocket of the LAC‐CTC327 interaction system, the hydroxyl groups in Asp227 and CTC327 formed a hydrogen bond with a distance of 3.63 Å. The *π*–*π* stacking interaction was established between the parallel benzene rings of CTC327 and Phe183. In addition to the hydrogen bond and *π*–*π* stacking interactions, hydrophobic interactions were observed between the hydrophobic groups of LAC (Pro412, Phe286, Phe353, and Phe183) and the benzene ring of CTC327 (Figure 4b). The RMSD graph shows stable interactions between LAC and CTC327 (Figure 4e). The gmx_MMPBSA tool calculated binding free energy of the LAC‐CTC327 was −137.851 KJ mol^−1^, suggesting its high stability (Table S7, Supporting Information). Asp228, Asp248, Ile250, Gln251, Pro278, Pro310, Pro313, Pro408, and Thr444 exhibited the significant effect on the intermolecular interaction of LAC‐CTC327, with Pro313 having the greatest negative energy (Figure 4f). From the results of molecular docking analysis, a possible electron transfer route during the CTC oxidation process mediated by SACS is proposed in Figure 4g. First, an electron was transferred from CTC327 to the T1 Cu of LAC, leading to the formation of the phenoxy radical (PhO**·**). After that, T1 Cu transferred the electrons via the Cys‐His pathway to the trinuclear clusters consisting of T2 Cu and two T3 Cu atoms, where the electrons were collected until four electrons were gathered. T3 Cu atoms then transferred the electrons to T2 Cu, and an O_2_ molecule reached this metal ion via an oxygen channel and was reduced into H_2_O, accompanied by the formation of hydroxy radicals. The delocalization of the unpaired electron of the radical intermediate would enhance its chance of interacting with the O—H bond of CTC via the H‐abstraction route,^[^ ^21^ ^]^ which consequently stimulates the catalytic cycle and boosts the remediation efficiency. The proposed electron transfer route was confirmed using EPR spectra (Figure S2, Supporting Information).

### 2.4 Catalytic Performance of Self‐Amplifying Catalytic System for Bioremediation

**Figure 5:**
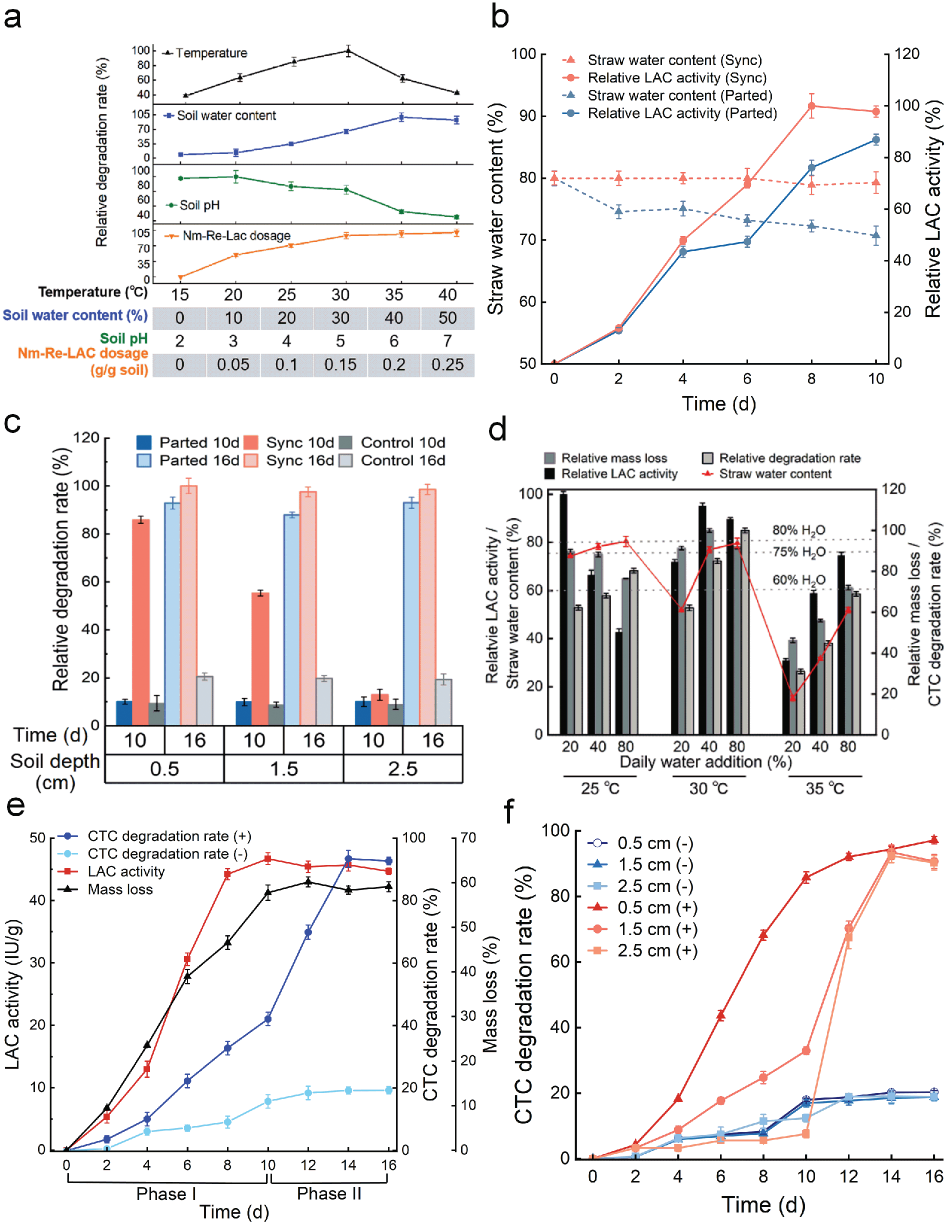
Coupling degradation of straw and chlortetracycline (CTC) in soil mediated by self‐amplifying catalytic system (SACS). a) Optimization of SACS for CTC removal in soil. b) Changes in laccase (LAC) activity and straw water content of SACS during the coupling process under the Synchronous model (Sync) and the Parted model (Parted). c) CTC remediation performance of SACS at various soil depths under Sync and Parted. The soil sample without Nm‐Re‐LAC treatment was designated as the control. d) Effects of daily water addition on the straw water content, straw mass loss, LAC activity, and CTC degradation during the coupling process. e) LAC production, straw degradation, and average CTC degradation during the modified coupling process. f) CTC degradation at various soil depths under the modified coupling process. Error bars represent S.D. (*n* = 3).

To modify the SACS for efficient CTC removal in the soil, the effects of five key factors (temperature, soil water content, soil pH, Nm‐Re‐LAC dosage, and Nm‐Re‐LAC implement approach) on CTC degradation rate were studied. As presented in **Figure** **5**a, the degradation rate was found to increase with the temperature from 15 to 30 °C. An obvious decrease in degradation rate occurred when the temperature was up to 35 °C due to reduced LAC activity and increased water loss. Soil moisture also played a significant role, with the proper moisture level found to be between 40% and 50% (Figure 5a). At higher moisture levels, oxygen availability was limited, and the CTC degradation rate decreased. The relative degradation rates were kept above 80% at a pH varying from 2.0 to 5.0 with an optimal pH of 3.0. Subsequently, although the CTC degradation rates decreased when the pH continued to rise, SACS still presented bioremediation capability (Figure 5a). Our previous work has demonstrated that the suitable pH range for enzyme production and straw degradation by CTrAT under SSF of straw was around 4.0–6.0 (Table S4, Supporting Information). Taking the efficiencies of CTC bioremediation, enzyme production, and straw degradation into account, the pH was set at 4.0 during the follow‐up coupling experiments. Nm‐Re‐LAC dosage was found to promote CTC degradation significantly up to 0.15 g/g soil, but a further increase in the dosage contributed little to the degradation rate (Figure 5a). The implementation approach of mixing Nm‐Re‐LAC into the soil achieved high remediation efficiency at various soil depths, while loading Nm‐Re‐LAC on the soil surface was only suitable for shallow soil contamination (Figure S3, Supporting Information).

In addition to soil bio‐remediation, the SACS based on Nm‐Re‐LAC could achieve simultaneous secretion of multiple lignocellulose‐degrading enzymes, namely endoglucanase, *β*‐glucosidase, xylanase, and LAC, enabling direct degradation of lignocellulosic biomass such as straw. Therefore, SACS has great potential for the coupled degradation of CTC in soil and straw.

To simulate agricultural practices, two models (synchronous and parted) were employed to investigate the capability of SACS to couple the degradation of CTC in soil and straw. The parted model, meaning that the CTrAT‐inoculated straw was placed on a plastic sheeting shell to obtain the Nm‐Re‐LAC. The synchronous model (Sync) refers to placing the straw inoculated with CTrAT directly onto the CTC‐contaminated soil. After 10 days of cultivation, the Nm‐Re‐LACs obtained from both models were blended into the soil. As shown in Figure 5b, the water content was maintained at ≈80% in the Sync model because the soil had a strong water‐holding capacity. In comparison, for the parted model, the water content decreased to 70.74% on day 10. In addition, the LAC activity in the Sync model was higher than that in the parted mode.

To analyze the soil remediation efficiencies of the two models using SACS, the CTC degradation rates at different soil depths were measured on days 10 and 16. As shown in Figure 5c, under the Sync model, the CTC in shallow soil depths were able to be remediated during Phase I (0–10 days), reaching 85.92% and 55.36% at 0.5 and 1.5 cm, respectively, on day 10, while those of the parted group and control group were lower than 10% in this phase. Although the degradation rates of the parted group increased significantly in Phase II (10–16 days) when Nm‐Re‐LAC was blended into the soil, reaching 92.88%, 87.93%, and 93.01% at 0.5, 1.5, and 2.5 cm, respectively, on day 16, the Sync model still exhibited better remediation performances with the degradation rates above 97% at all soil depths tested. Taken together, the Synchronous model had better performance in holding water and enzyme production, thus leading to a better remediation performance in the coupled degradation of CTC in soil and straw.

### 2.5 Coupling of CTC‐Contaminated Soil Remediation and Straw Degradation via SACS

The key parameters of SACS for the coupled degradation of CTC in soil and straw were studied. Water supplementation was critical for straw degradation and soil remediation because the coupling process was performed in the open air. It is important to mention that the amount of water added may vary depending on the specific ambient temperature because a higher temperature could enhance water volatilization from both straw and soil, and affect the water‐holding capacity of the straw matrix. Therefore, three levels of daily water addition (20%, 40%, and 80%) were applied under temperatures ranging from 25–35 °C. At a fixed temperature, an increase in water supplementation enhanced the water content of the straw matrix (Figure 5d). It is noteworthy that the changes in water supplementation did not bring obvious differences in water content when the temperature was set at 25 °C, suggesting that the water‐holding capacity of the straw matrix was close to its upper limit at this temperature, and a further increase in daily water addition hardly enhanced the water content of the straw matrix.

According to our previous research, the optimum water content of the straw matrix for LAC and cellulase production by CTrAT was 75%, and the consortium maintained high enzyme production capability when the straw water content was within the range of 60–80% (Table S4, Supporting Information). Therefore, in this study, at a fixed temperature, the LAC activity was higher when the water content was closer to 75% (Figure 5d). For instance, the maximum LAC activity at 25, 30, and 35 °C was achieved when daily water addition was 20%, 40%, and 80%, respectively. Given that the activities of LAC and cellulase are the two key factors governing straw degradation, similar results could be observed with regard to straw biomass loss. As shown in Figure 5d, at a fixed temperature, when the straw water content was close to 75%, biomass loss reached the maximum at the corresponding water supplementation. However, the inhibition of straw degradation occurred when the temperature was increased to 35 °C, indicating that this temperature severely impaired the consortium's metabolism and enzyme production capability.

As for CTC degradation on the soil surface at a fixed temperature, the degradation rate increased with daily water addition, which could be associated with the higher leaching and diffusive transport of LAC. Additionally, when the straw water content was within a suitable range (60–80%), at a fixed daily water addition, the maximum CTC degradation rate was achieved at 30 °C which was consistent with the optimal temperature for CTC removal catalyzed by the SACS (Figure 5a). In this case, the suitable combination of daily water addition and temperature coupling degradation of CTC and straw was 80% at 30 °C. The results largely confirm our previous findings of the better performance of synchronous model in coupling degradation because it could maintain a straw water content of ≈80% (Figure 5b).

Soil cover over the straw matrix should ensure moisture and temperature stability of the reaction system, and allow sustainable (though not necessarily) remediation. Ideally, a soil cover over the straw matrix should restrict water in the soil^[^ ^33^ ^]^ and thus maintain a suitable environment for the coupled degradation of straw and CTC mediated by SACS. In this study, two strategies were applied to evaluate the effect of soil cover on the coupling process at 30 °C. The first one was designated as “Cover,” meaning that the straw matrix was covered using a plastic film incorporating small pinholes, and meanwhile, daily water supplementation was eliminated in this group. The other was referred to as “Open,” meaning the straw matrix was supplemented with 80% daily water addition without a soil cover. Straw water content, LAC production, and CTC degradation rates at different soil depths were analyzed. According to the changes in the straw water content, both strategies could maintain a comparatively stable humidity range favorable for the coupled degradation (Figure S4a, Supporting Information). For Cover group, the application of soil cover effectively reduced water evaporation and maintained the water released by CTrAT's metabolism within the system. Without daily water supplementation, the water content in the straw matrix increased slowly instead of decreasing (Figure S4a, Supporting Information). As for changes in LAC activity, the two strategies showed similar LAC production processes, although the Cover group achieved higher LAC activity at the end of the coupling process. Similarly, after 16 days of coupled degradation, applying a soil cover did not result in obvious differences in the CTC degradation rates (Figure S4b, Supporting Information).

The coupling of straw degradation and CTC‐contaminated soil bioremediation mediated by SACS was studied using a modified coupling process. The changes in the LAC activity, straw mass loss, and average CTC degradation rate during the coupling process are shown in Figure 5e. Nm‐Re‐LAC, produced during Phase I of the coupling process, could secrete several enzymes, including LAC. LAC activity had been increasing rapidly during the first 8 days, reaching 46.7 IU g^−1^ on day 10. Nm‐Re‐LAC initiated CTC degradation and released the active mediator, CTC327, which in turn boosted the catalytic capability of Nm‐Re‐LAC, promoting the conversion of CTC into CTC327, thus forming a self‐amplifying catalytic system. As a result, during Phase II, when Nm‐Re‐Lac was blended into the soil, rapid degradation of CTC was observed, and the average CTC degradation rate reached 93.43% with a straw mass loss of 58.35% on day 14 (Figure 5e).

For CTC remediation at different soil depths in Phase I, CTC degradation, mainly mediated by SACS, was conducted at shallow soil depths. During this period, LAC began seeping into the soil along with water and slowly degraded CTC with a degradation rate of 85.78% at a soil depth of 0.5 cm on day 10 (Figure 5f). However, because of the limited amount of LAC seeping into the water via this path, the CTC degradation rate decreased significantly with the soil depth and was less than 10% at a soil depth of 2.5 cm on day 10. In Phase II, in addition to the thoroughly mixed Nm‐Re‐Lac, persistent evapotranspiration could cause the upward migration of the contaminant, and thus CTC degradation rate began to rise, reaching 94.39%, 93.48%, and 92.42% at the soil depth of 0.5, 1.5, and 2.5 cm on day 14, respectively.

We believe that SACS can achieve effective degradation of straw and remediation of CTC‐contaminated soil via a coupled process and can be used in organic pollutant degradation for environmental remediation and sustainable agricultural practices.

## 3 Discussion

Redox mediators have been recognized as key factors for LAC‐catalyzed bioremediation, especially when correlated with pollutants of higher redox potential (>0.8 V), because the mediators can act as electron shuttles between the substrate and the enzyme, thus improving the oxidative capability of LAC. The SACS reported here exhibits a key feature desired for large‐scale LAC‐based bioremediation processes, such as redox mediator regeneration (Figure 1). In search of clues as to how this was achieved, this work first reports the molecular interactions between the potential mediators and LAC in the key catalytic core (T1 Cu). A novel mediator, CTC327, derived from CTC degradation, was identified with greater affinity and stronger molecular bonds to the T1 Cu site than existing mediators, including the widely used mediator HBT (Figure 4 and Table S3, Supporting Information). The binding event between the mediator and LAC plays a decisive role in the oxidation reaction. An appropriate mediator can facilitate electron transfer at the T1 Cu site by creating a suitable environment for the positioning of the substrate and the progress of the reaction.^[^ ^34^ ^]^ In this way, instead of binding to the active cavity of LAC, the substrate (i.e., CTC) can transfer electrons to the active mediator CTC327 to initiate oxidation, which facilitates electron transfer between LAC and the substrates, and consequently boosts remediation efficiency. This corroborates the high efficiency of TC degradation in the soil mediated by SACS. Biodegradation has emerged as an effective method for the removal of TCs from aqueous environments.^[^ ^35^, ^36^, ^37^, ^38^ ^]^ However, information regarding TC biodegradation in soil remains limited. A bacterial consortium comprising *Pandoraea* sp. and *Raoultella* sp. showed an increased TTC degradation efficiency compared to a single strain, with the TTC concentration decreased by 43.72% within 65 days.^[^ ^39^ ^]^ Shi et al. explored the OTC removal in soil using *Arthrobacter nicotianae*, and observed an increase in OTC degradation by 8.22–45.45%.^[^ ^40^ ^]^ It was reported that arbuscular mycorrhizal fungi can enhance OTC decomposition and reduce residual OTC concentrations.^[^ ^41^ ^]^ Besides, the addition of manure slurry to soil was revealed to increase the microbial population or activity and promote the degradation efficiency of sulfadimethoxine.^[^ ^42^ ^]^ SACS‐mediated transformation of TCs is of great interest because it is a low‐cost as well as environmentally friendly remediation approach. Furthermore, compared to other LAC‐mediator systems, SACS achieves efficient removal of CTC without the addition of any mediators. As shown in Figure 1, the lignin‐derived natural mediators from Nm‐Re‐LAC initiate CTC degradation. Subsequently, CTC327 starts the self‐amplifying catalytic cycle, and the in situ regenerated active intermediate acts as a mediator for LAC, enabling a chain reaction for highly effective CTC degradation.

Our results also provide insights into the treatment of agricultural waste mediated by the fungal consortium. Cellulosic biomass is resistant to degradation because of its complex and rigid structure. Lignin plays a negative role in deconstruction, and efficient lignin removal can significantly improve the enzymatic digestibility of biomass.^[^ ^43^ ^]^ White‐rot fungi are of particular interest owing to their ability to produce highly active LACs using lignocellulosic residues as substrates during SSF, which allows for simultaneous LAC production and lignin degradation.^[^ ^44^, ^45^, ^46^ ^]^ Previous studies have investigated various methods to enhance LAC production by white‐rot fungi.^[^ ^47^, ^48^, ^49^, ^50^, ^51^, ^52^ ^]^ For example, Lonappan et al. used various inducers, such as veratryl alcohol and phenol red, to enhance LAC production by *T. versicolor*, a maximum LAC activity of 49.16 and 14.26 U g^−1^ was obtained from apple pomace and alfa plant fibers, respectively.^[^ ^47^ ^]^ LAC production by *T. versicolor* of 25.7 U g^−1^ dry substrate was achieved using tea residues.^[^ ^48^ ^]^ The combination of syringic acid and sweet sorghum bagasse led to an enhanced LAC activity of 67.4 U g^−1^ on day 16 by *Coriolus versicolor*.^[^ ^49^ ^]^ The utilization of *Eichhornia crassipes* by *P. sanguineus* was shown to produce LAC with an activity of 7.26 U g^−1^ dry substrate.^[^ ^50^ ^]^

Although research efforts to increase LAC production by white‐rot fungi have been made, the degradation rates of cellulose, hemicellulose, and lignin in lignocellulosic substrates remain low after fungal pretreatment.^[^ ^53^ ^]^ Recent studies have suggested that the co‐culture of LAC‐producing strains with other microorganisms can offer unique advantages, such as higher LAC yields and more desirable properties for disrupting recalcitrant lignocellulosic structures.^[^ ^51^, ^52^ ^]^ In this study, recombinant *T. reesei*, which can secrete *P. sanguineus* LAC, together with *T. versicolor* and *A. niger*, comprised a biodiversity of LAC‐producing species. The resulting diverse sets of LACs exhibited encouraging results in terms of efficient lignin degradation. Compared with previous studies, efficient LAC production with a higher lignin degradation rate (81.64% on day 10) was achieved using CTrAT.

Moreover, the co‐culture consortium can enhance the interactions between microorganisms and thus compensate for the deficiency in enzyme production performances of the specific strain.^[^ ^54^ ^]^ Besides LAC, the marked increases in lignocellulose‐degrading enzyme production by CTrAT were also observed, such as *β*‐glucosidase, endoglucanase, and xylanase, which led to improved synergistic hydrolysis of the substrate (straw). For instance, the *β*‐glucosidase contributes to the removal of accumulated cellulose solubilization products (i.e., cellobiose) and the feedback inhibition of the enzyme activities of other components in the cellulase complex.^[^ ^24^ ^]^ Thus, CTrAT—capable of secreting a variety of cellulolytic enzymes—can fully alleviate the recalcitrance of straw, especially the lignin, for the effective deconstruction of lignocellulose (Figure 2).

Based on CTrAT, the resulting koji (Nm‐Re‐LAC) formed during the straw degradation process was abundant in lignin‐derived natural mediators coupled with high‐activity LAC and, as described previously, was employed in launching the CTC degradation. Compared with conventional artificial mediators for LAC (such as ABTS and HBT), natural mediators, which are phenolic compounds generally associated with lignin decomposition, are environmentally friendly substitutions with lower costs.^[^ ^21^ ^]^ Furthermore, notable increases in TCs under the coexistence of different types of natural mediators (Table S1, Supporting Information), potentially through a cooperative effect caused by chain reactions among multiple radical species,^[^ ^21^ ^]^ highlighted the importance of Nm‐Re‐LAC in high‐efficiency bioremediation. Overall, this study demonstrated an a mediator and effective catalyst derived from resource‐abundant and renewable biomass, providing new insights into organic pollutant degradation and diverse applications for environmental sustainability.

## 4 Experimental Section

### Materials, Strains, and Chemicals

*T. reesei* ZJ09, harboring the *P. sanguineus* LAC gene, was constructed in the previous study.^[^ ^19^ ^]^ *A. niger* was stored at the Laboratory of Biochemical Engineering of Zhejiang University. *T. versicolor* was obtained from the New Zealand Timber Anticorrosion Research Center. The strains were preserved on potato dextrose agar (PDA) at 4 °C. The inoculum was prepared according to the previously described.^[^ ^55^ ^]^ Antibiotics standards (99% purity) and mediators were purchased from Sigma‐Aldrich.

Rice straw was collected from a suburban district in Hangzhou, Zhejiang Province, China. For pretreatment, straw samples were cut into 2–3 cm and saturated with ammonium hydroxide for 24 h as previously described.^[^ ^56^ ^]^

### Inoculum Preparation

The three strains were cultured on PDA medium for 5–7 days at 30 °C. For *T. reesei* and *A. niger*, sterile saline (7–9 mL) was pipetted into fully sporulated slants to prepare spore suspensions (1 × 10^8^ spores per mL) that were used as inoculum. For *T. versicolor*, disks of mycelia (5 mm‐diameter) were cut from the fully grown slants, followed by the addition of 50 mL of the inoculum medium, which consisted of: yeast extract 1.0 g L^−1^, glucose 10.0 g L^−1^, CaCl_2_ 0.3 g L^−1^, KH_2_PO_4_ 2.0 g L^−1^, (NH_4_)_2_SO_4_ 1.4 g L^−1^, urea 0.3 g L^−1^, and MgSO_4_·7H_2_O 0.1 g L^−1^. The pH was adjusted to 4.8. *T. versicolor* was cultured at 30 °C for 7 days as inoculum.

### Straw Degradation and Nm‐Re‐LAC Preparation

Rice straw degradation by the fungal consortium was performed in 500 g capacity plastic trays (length of 11 cm, width of 8 cm, and height of 8.5 cm). Hundred grams of a total dry straw was mixed with 250 mL SSF nutrient solution,^[^ ^55^ ^]^ and the degradation process was carried out with a substrate water content of 75% at pH 4.0, at 30 °C for 10 days according to Tables S4–S6, Supporting Information.

For straw degradation by Tr–An, *T. reesei* spore suspension was inoculated into the trays from the beginning (designated as D0). *A. niger* was inoculated on D2, 2 days after the inoculation of *T. reesei* with an *A. niger* to *T. reesei* ratio of 1:5 v/v. For straw degradation by Tr–Tv, *T. versicolor* was inoculated on D1, 1 day after inoculation with *T. reesei* at a *T. versicolor* to *T. reesei* ratio of 1:1 v/v. For straw degradation by An–Tv, *T. versicolor* and *A. niger* were inoculated on D0 and D1, respectively, at a 1:5 v/v ratio. For straw degradation by CTrAT, *T. reesei*, *T. versicolor*, and *A. niger* were inoculated on D0, D1, and D2, respectively, at a ratio of 1:1:5 v/v/v.

The total inoculum size was 10% v/w among all groups, and the straw substrate was sampled every 2 days for enzyme assays and determinations of straw components. The koji obtained from SSF using CTrAT for 10 days was designated as Nm‐Re‐LAC and was taken for antibiotic degradation and CTC‐contaminated soil remediation.

### Soil Collection and Preparation

Soil samples were collected from Yuhang District, Hangzhou City, Zhejiang Province, China. The collection of soil was performed as previously described.^[^ ^57^ ^]^ To simulate CTC‐contaminated soil, 5.0 kg of soil was added into a plastic tray (length of 30 cm, width of 20 cm, and height of 25 cm), and CTC in methanol (500 mg L^−1^) was evenly poured into the soil and mixed several times. The CTC extracted from the soil was used as the initial CTC concentration (≈80 mg kg^−1^) in the soil remediation experiments.

### Degradation of Tetracycline Antibiotics Solution

Degradation of TCs (CTC and OTC) was performed using Nm‐Re‐LAC or crude laccase (LAC‐ZJ09). LAC‐ZJ09 was prepared using recombinant *T. reesei* ZJ09 under submerged fermentation, following previously described procedures.^[^ ^19^ ^]^ A mixture of 80 mg L^−1^ of CTC or OTC was treated with either Nm‐Re‐LAC containing 0.2 IU mL^−1^ LAC or LAC‐ZJ09 with or without mediators. The concentration of a single mediator was 0.3 mm. For the syringaldehyde/vanillin complex, 0.3 mm of each mediator was mixed at a Syr to Van proportion of 4 to 6. The reaction was carried out for 4 h at 50 °C and at a pH of 4.0. Residual antibiotic concentrations were determined by high‐performance liquid chromatography (HPLC) (see the “Analysis” section for details).

### Molecular Docking and Dynamics Simulations for LAC‐Mediator Complexes

The crystal structure of *P. sanguineus* LAC (PDB accession code: 5NQ7) was obtained from the Protein Data Bank. Binding free energies and potential binding sites were explored by AutoDock suite 4.2.6.^[^ ^58^ ^]^ The docked conformations were visualized by PyMOL, and the non‐covalent interactions between LAC and mediators were identified using the PLIP web server.^[^ ^59^ ^]^ The docked complex with the lowest score was selected for further dynamics simulations.

The dynamics simulation was performed using the GROMACS software (version 2020.3). All simulations were performed for 25 ns at a temperature of 300 K and a pressure of 1 atm. The van der Waals force was calculated via Lennard–Jones function, with the non‐bond truncation distance of 1.4 nm. Visual molecular dynamics was used to analyze the trajectory, and gmx_MMPBSA (http://jerkwin.github.io g^−1^ mxtool) was used to calculate molecular mechanics Poisson–Boltzmann surface area.

### Optimization of CTC Degradation in Soil by SACS

The key parameters including the koji implementation approach (Cover or Mix), koji dosage (0–0.25 g/g soil), temperature (15 to 35 °C), pH (2.0–7.0), and soil water contents (10–50%) were optimized. Five variables were analyzed individually. The condition that achieved the highest CTC degradation rate was subsequently applied in the optimization experiment for the next factor, while the other factors remained unchanged. For the optimization of the first parameter, temperature, CTC‐contaminated soil (250 g) was added to a 500 g capacity plastic tray. The Nm‐Re‐LAC was mixed with the soil at a dosage of 0.15 g/g soil. The degradation experiment was carried out from 15–40 °C, at a pH of 4.0, and with the soil water content of 40%, in an incubator for 3 days. Soil was sampled using a five‐point sampling method.^[^ ^60^ ^]^ The antibiotic biodegradation levels were determined using HPLC (see the “Analysis” section for details).

### Coupling Degradation of CTC‐Contaminated Soil and Straw

Coupling degradation was performed via two phases (Phase I and Phase II) in a 500 g capacity plastic tray with 250 g of CTC‐contaminated soil. Straw (25 g) was spread on the soil to a height of 2–3 cm. CTrAT at 10% v/w was inoculated into the trays. The pH of the soil was adjusted to 4.0 using citrate buffer. Unless otherwise stated, 80% daily water was added to the straw matrix using citrate buffer (pH 4.0). During Phase I (0–10 days), two models (parted and synchronous) were applied. For the parted model, the inoculated straw matrix was placed on a shell of plastic sheeting for 10 days to obtain Nm‐Re‐LAC. For the synchronous model, the inoculated straw was placed directly on the CTC‐contaminated soil for 10 days to obtain Nm‐Re‐LAC. On day 10, the Nm‐Re‐LACs formed from both strategies were blended into the soil, and the coupling process was conducted for another 6 days under the same conditions (Phase II). The straw matrix and soil were sampled every 2 days using a five‐point sampling method^[^ ^60^ ^]^ to determine LAC activity, straw mass loss, and CTC degradation rate.

### Analysis

Compositional analyses of the straw samples were performed using the National Renewable Energy Laboratory protocol.^[^ ^61^ ^]^

Citrate buffer (50 mm, pH 4.8) was used for the enzyme extraction of Nm‐Re‐LAC at a solid‐to‐liquid ratio of 1:5. The extraction process was performed by shaking at 30 °C, 200 rpm for 60 min, and then centrifuged at 4 °C, 10 000 × *g* for 5 min. The obtained supernatant was used for enzyme assays. Assays for LAC activity, FPA, BGA, CMCase activity, and xylanase activity were performed as previously described.^[^ ^55^ ^]^ The “enzyme activity” represents the mean value of three independent replicates, and “relative enzyme activity” in each set refers to the proportion of the enzyme activity to the highest activity in this set.

Antibiotic concentrations were determined using HPLC (Agilent 1200, USA) according to Xia et al.,^[^ ^55^ ^]^ except that the mobile phase for CTC was composed of 0.01 m oxalic acid, acetonitrile, and methanol (10:2:2, v/v/v).

The CTC degradation products were identified using HPLC/ESI‐MS acquired by Agilent 6545 Q‐TOF mass spectrometer (Agilent Technologies, USA). MS analysis of the intermediates was performed as previously described^[^ ^40^ ^]^ within a mass‐to‐charge (m/z) range of 150–700 m/z. Growth inhibition tests of *E. coli*, *B. subtilis*, and *P. subcapitata* by CTC and OTC after Nm‐Re‐LAC treatment were performed according to the methods previously described.^[^ ^62^ ^]^

## Conflict of Interest

The authors declare no conflict of interest.

## Author Contributions

Y.X. performed the experiments, analyzed the data, and wrote the manuscript; L.X. conceived the study and designed experiments; X.L. revised the manuscript and supervised the project. All authors discussed the results and commented on the manuscript.

## Supporting information

Supporting Information

## Data Availability

The data that support the findings of this study are available from the corresponding author upon reasonable request.
