# Supplementary material for: Laccase‐Based Self‐Amplifying Catalytic System Enables Efficient Antibiotic Degradation for Sustainable Environmental Remediation
Source: Adv Sci (Weinh). 2023 May 21;10(21):2300210. doi: 10.1002/advs.202300210 (PMC10375088; doi:10.1002/advs.202300210)
Supplement: Supplementary file 1 — Supporting Information [file ADVS-10-2300210-s001.pdf]

ADVANCED SCIENCE
Open Access

Supporting Information

for *Adv. Sci.*, DOI 10.1002/advs.202300210

Laccase-Based Self-Amplifying Catalytic System Enables Efficient Antibiotic Degradation for Sustainable Environmental Remediation

*Ying Xia, Liming Xia and Xinda Lin**

Supporting Information

Laccase-based self-amplifying catalytic system enables efficient antibiotic degradation for sustainable environmental remediation

Ying Xia[1], Liming Xia[2], Xinda Lin[1*]

[1]Key Laboratory of Bioorganic Synthesis of Zhejiang Province, College of Biotechnology and Bioengineering, Zhejiang University of Technology, Hangzhou, 310014, PR China

[2]Key Laboratory of Biomass Chemical Engineering of Ministry of Education, College of Chemical and Biological Engineering, Zhejiang University, Hangzhou, 310027, PR China

* Corresponding author: linxinda@zjut.edu.cn (X. Lin)

This supporting information file includes:

- Figure S1-S4
- Table S1-S7
- Methods

1. Electron paramagnetic resonance spectroscopy (EPR)
2. CTC extraction from the soil

∗Corresponding author. Tel./fax: +86 139 5802 8822.

E-mail address: linxinda@zjut.edu.cn (X. Lin).

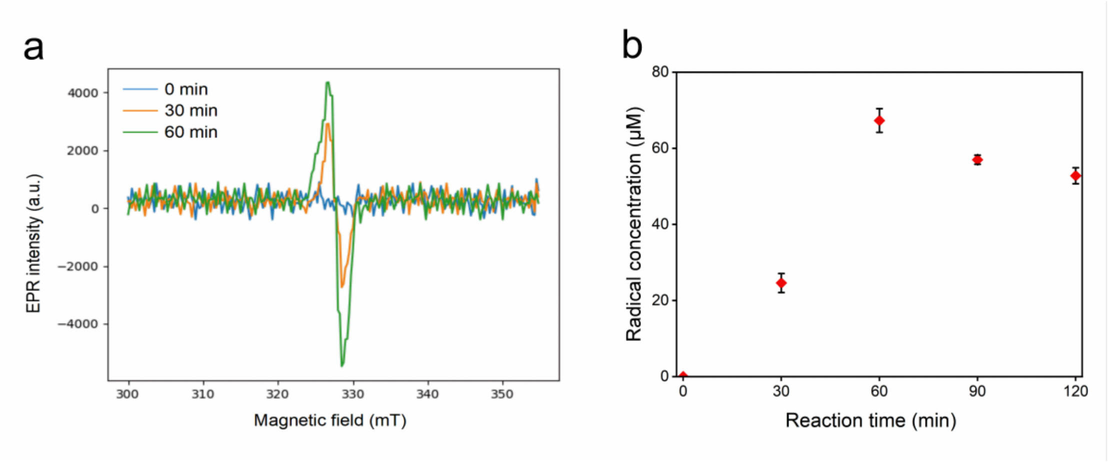

Figure S1. EPR analysis for radical formation during lignin degradation after treatment with LAC. (a) Overlay of EPR spectra with radical signal responses in lignin suspensions measured at varying time points. (b) Kinetics of radical formation during LAC action on lignin. Reaction (5 mL) was performed using 10% (w/v) lignin suspension with LAC dosage of 1 IU/mL. The stable nitroxyl radical TEMPO (0.5 to 100 μM) was used to convert the number of spins to a radical concentration.

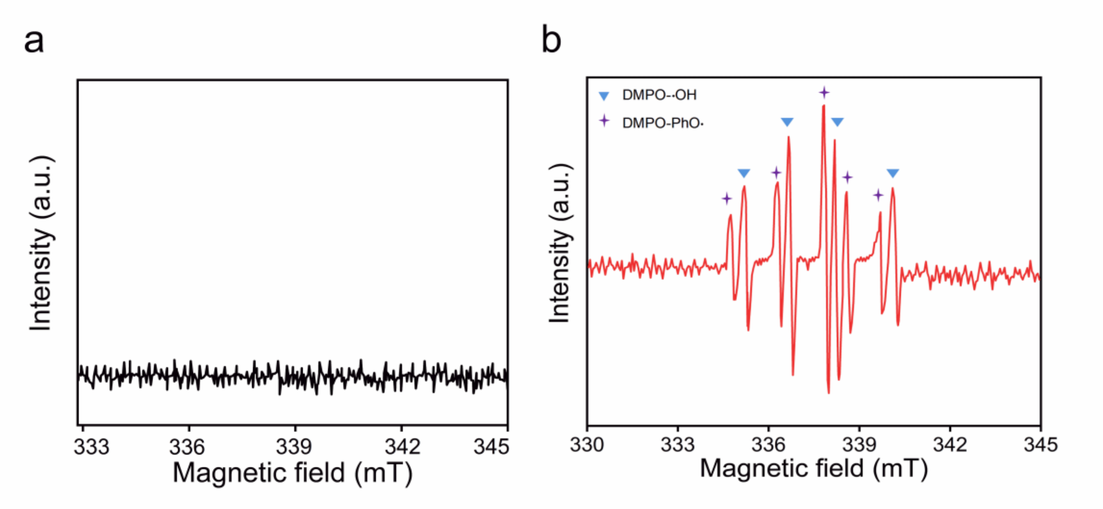

Figure S2. EPR spectra for radical formation during CTC degradation. (a) Spectra recorded at 0 min. (b) Spectra recorded at 30 min. The reaction (5 mL) was catalyzed by Nm-Re-LAC using 20 mg/L CTC with LAC dosage of 1 IU/mL.

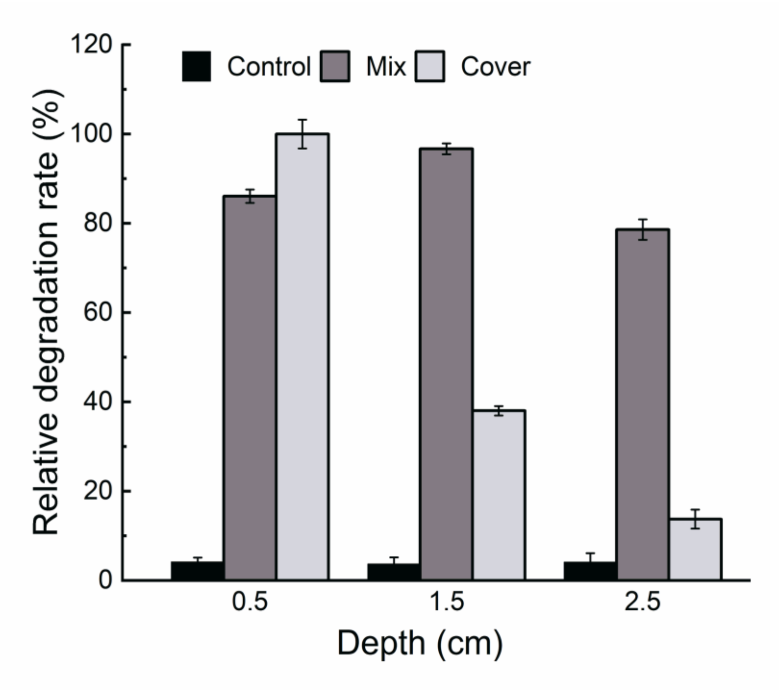

Figure S3. Effect of Nm-Re-LAC implementation approach on the degradation rate of CTC. Error bars represent s.d. (n = 3).

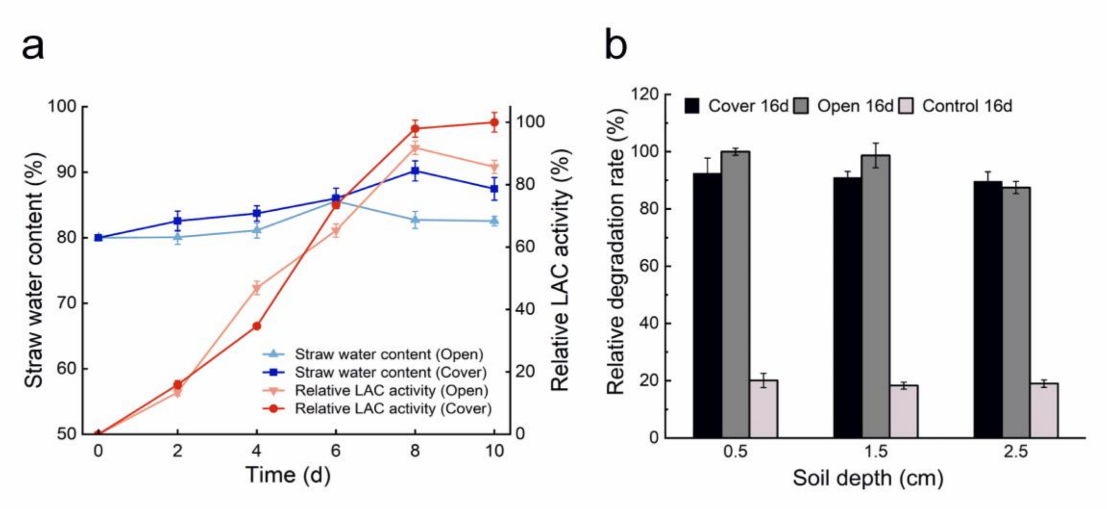

Figure S4. Study of soil cover on the SACS-mediated coupling process. (a) Effects of soil cover on the straw water content and LAC activity. (b) Effect of soil cover on CTC degradation. Error bars represent s.d. (n = 3).

Table S1. Degradation of tetracycline antibiotics by laccase-mediator systems.

Laccase-mediator system	OTC degradation rate (%)	CTC degradation rate (%)
Control	4.18 ± 0.09	5.67 ± 0.11
LAC[a] without mediator	10.73 ± 0.32	41.15 ± 0.84
LAC[a]+ABTS	46.73 ± 0.83	86.10 ± 0.97
LAC[a]+HBT	30.31 ± 0.79	90.25 ± 1.13
LAC[a]+Syringaldehyde	62.98 ± 1.04	71.43 ± 1.02
LAC[a]+Vanillin	61.54 ± 0.93	75.80 ± 0.89
LAC[a]+Syr/Van	90.52 ± 1.18	82.03 ± 0.92
Nm-Re-LAC[b]	89.12 ± 1.07	95.69 ± 1.04

[a] LAC-ZJ09 refers to crude laccase produced by the *T. reesei* ZJ09[1] under submerged fermentation.

[b] Nm-Re-LAC refers to the natural mediator-regenerating koji with high laccase activity, which was obtained from SSF of straw by fungal consortium constructed in this study (see the "Straw degradation and Nm-Re-LAC preparation" section for details).

Table S2. Growth inhibition towards bacteria and algae caused by tetracycline antibiotics after Nm-Re-LAC treatment.

Antibiotics	Treatment	Growth inhibition (%)[a]		
	Time (h)	*B. subtilis*	*E. coli*	*P. subcapitata*
CTC	0	100.0 ± 0.3	97.3 ± 0.9	91.5 ± 2.1
	0.5	4.2 ± 0.4	6.7 ± 3.1	6.3 ± 0.8
	1.0	0	0	0
OTC	0	98.1 ± 3.6	96.2 ± 4.1	81.7 ± 3.4
	0.5	9.5 ± 0.8	7.2 ± 1.9	8.3 ± 1.2
	1.0	0	0	0

[a] Values represent means±standard deviations (n=3).

Table S3. Comparison of LAC-mediator complex geometry at T1 Cu site.

Laccase-mediator complex	Affinity (kcal/mol)	Distances (Å)[a]

LAC-HBT	-4.8	7.0
LAC-Syr	-4.2	7.4
LAC-Van	-3.8	7.7
LAC-CTC	-1.0	10.6
LAC-CTC430	1.5	7.2
LAC-CTC406	-2.6	6.7
LAC-CTC327	-6.4	6.5
LAC-CTC284	-6.0	7.3

[a] Average distance between the nuclei of T1 Cu and atoms in mediators.

Table S4. Effect of substrate water content on enzyme production and straw degradation by CTrAT under SSF on day 10.

Substrate water content (%)	Relative LAC activity (%)	Relative FPA (%)	Relative xylanase activity (%)	Relative mass loss (%)
55	79.63	84.45	59.97	87.07
60	82.30	91.93	84.75	89.65
65	84.95	94.64	100.00	92.31
70	86.05	93.88	87.90	95.67
75	100.00	100.00	82.74	100
80	91.79	93.04	81.17	87.23

Table S5. Effect of pH on enzyme production and straw degradation by CTrAT under SSF on day 10.

pH	Relative LAC activity (%)	Relative FPA (%)	Relative xylanase activity (%)	Relative mass loss (%)
3	62.91	93.45	73.74	98.72
4	100.00	94.64	90.52	100
5	92.30	94.93	91.75	99.67
6	84.05	97.88	100.00	97.65
7	63.43	100.00	89.74	97.91

8	25.72	79.96	73.90	81.42

Table S6. Effect of temperature on enzyme production and straw degradation by CTrAT under SSF on day 10.

Temperature	Relative LAC activity (%)	Relative FPA (%)	Relative xylanase activity (%)	Relative mass loss (%)
20	75.72	69.47	53.57	89.35
25	100.00	88.42	64.28	100.00
30	89.19	100.00	92.82	97.14
35	58.14	71.37	100.00	92.94

Table S7. MMPBSA analysis of LAC-CTC 327.

Energy	LAC-CTC 327
van der Waals energy(KJ/mol)	-87.193
Electrostatic energy (kJ/mol)	-15.962
Polar solvation energy (KJ/mol)	48.112
Nonpolar solvation energy(KJ/mol)	-52.886
Total binding energy(KJ/mol)	-157.929
TΔS(KJ/mol)	20.078
Total binding free energy(KJ/mol)	-137.851

Methods

1. Electron paramagnetic resonance spectroscopy (EPR)

EPR analysis of CTC treated with Nm-Re-LAC for radical investigation was performed as previously described[2]. To conduct the test, 50 μL of the suspension was taken for immediate detection, with the following settings: Modulation amplitude of 0.2 mT, sweep time of 30 s, and sweep width of 10 mT. The measurement was conducted at 20 °C with a MiniScope MS200 (Magnettech, Berlin, Germany). The area under the absorption signal was converted to a radical concentration by a linear standard curve based on the solutions of stable nitroxyl radical, TEMPO, with concentrations of 0.5–100 μM.

2. CTC extraction from the soil

CTC extraction from the soil was according to the previously reported method[3]. Briefly, 1 g of soil sample was transferred to 50 mL centrifuge tubes with 10 mL of EDTA-Mcllvaine buffer (pH = 4.0). The mixture was then vortexed for 1 min, ultrasonically extracted for 30 min, and centrifuged for 5 min at 6500 × *g*. The supernatants were collected, while the sediment was treated repeatedly under the same process. The obtained supernatants were filtered with a 0.45 μm fiber filter and pumped into a 6 mL Oasis HLB cartridge (500 mg, Waters) at a flow of 5 mL/min. After that, the cartridge was washed using 15 mL of ultrapure water and eluted with 8 mL of methanol containing 0.1% formic acid (v/v).

References

[1] J. Zhao, S. Zeng, Y. Xia, L. Xia, *J. Biosci. Bioeng.* **2018**, *125* (4), 371.
[2] L. Munk, M. L. Andersen, A. S. Meyer, *Enzyme Microb. Technol.* **2018**, *116*, 48.
[3] H.-Y. Liu, C. Song, S. Zhao, S.-G. Wang, *Environ. Sci. Technol.* **2020**, *706*, 136086.
